# Eye Diseases and Other Reflexes, Due to Errors of Refraction

**Published:** 1887-12

**Authors:** T. J. Tyner

**Affiliations:** Austin, Texas


### EYE DISEASES, AND OTHER REFLEXES, DUE TO ERRORS OF REFRACTION.

READ BEFORE THE AUSTIN DISTRICT MEDICAL SOCIETY, AND BY VOTE ORDERED PUBLISHED IN THIS, THE OFFICIAL ORGAN OF THE SOCIETY.

*T. J. Tyner, M. D., Austin, Texas.*

THE object of this paper is to call your attention to the importance of early recognizing the errors of refraction in the many cases of eye diseases which you are called upon to treat. I would not have you think that I put a pair of correcting glasses on every patient with the slightest refractive error, for, in reality, there are but few, comparatively speaking, who are theoretically and absolutely emetropic, and to judiciously prescribe a correcting glass requires good judgment and a thorough history of the case.

Myopia always exist when the retiua is beyond the refractive system of the eye, i. e., when the antero posterior diameter is too long to focus parallel rays on the retina. If not of too high a degree, myopia does not constitute a troublesome defect. On the contrary, it may be advantageous under certain circumstances. It is generally acquired, for we know that the emetrope, and even the hypertropes of low degree may develop into myopia particularly in the very studious children may be considered as a provision of nature, to adapt the organ to the conveniences and functions of a superior race. It is a well known and an interesting fact, that all animals are hypermetropic, myopia being peculiar to the human race, and moreover, that it is much more frequently met with among civilized nations, than savages. Hence, we may conclude that myopia has developed in the human race, and the determining cause is civilization, and it would not be unreasonable to predict that ultimately, when civilization has reached its highest standard, the human race will be one of myopia, i. e., myopia will prevail over other errors.

Of the two anomalies, hypermetropia and myopia, the latter is the least, for the hyperopic eye, without an effort of the accommodation or correcting glasses, is not adapted to any distance, hence, is absolutely too short, while the myopia eye is too long only for great distances, but is perfectly adapted to near vision. There being but little or no accommodative effort in myopia, asthenopic symptoms are rarely ever met with, and unless it is of high degree, glasses, even, are seldom required, except for distance. In the short, or hypermetropic eye, it is very different. As has already been stated, the hypermetropic eye is too short, and when in a state of rest, is not adapted to any diatance, for the reason the principal focus of the refractive apparatus lies behind the retina, and in such an eye perfect vision can only be obtained by an effort of the accommodation. It is this effort or strain that gives rise to the symptoms known as asthenopia. During close work the ciliary muscle is in a constant state of contraction, and after a time becomes relaxed, owing to fatigue, when letters, or near objects, become blurred and indistinct, and the patient must close the eye, or look in the distance until the muscle regains its functions. It is surprising to see the difference in personal susceptibility. In many, high degrees of hypermetropia, and even astigmetism are born without the patient even being aware of any inconvenience.

Some young hypermetropes have such an amplitude of accommodation, that they have perfect vision for both distant and near objects, and would no more tolerate a correcting glass than would an emetrope, and it is not until they grow older, or have some debilitating disease that their error manifests itself. Again, in others, the slightest error causes the most serious annoyance, and all work at near objects must be abandoned, or finally some inflammatory disease is developed. The most frequent of these are hordeolum, or stye, which appears from time to time, until the integrity of the eyelids is threatened. Bapharitis and the various forms of anqurativitis. These inflammatory troubles are always obstinate, and sooner or later, assume a chronic character. Other symptoms are headache, ciliary and supra-orbital neuralgias, hyperesthesia of retina and photophobia. Hypermetropia is also supposed to be a factor in glaucoma, which is explained by the fact that the eye is smaller, with a more unyielding sclera than the myopic or emetropic eye. By these symptoms hypermetropia is easily recognized. The sufferer will tell you that he can see well at a distance, and so long as he refrains from near work, is quite comfortable, but has difficulty in fixing near objects for any length of time, and if he has been compelled to use his eyes at near work, will most likely present some of the inflammatory symptoms above mentioned,

Young children who are anæmic, or recently recovered from diphtheria, scarlet fever, or some debilitating disease, suffer the same annoyance as old persons. It is not out of place here to mention that, as a rule, myopic children are much more studious than the hyperopic, for the reason, no doubt, that he does so without any inconveniences, i. e., because he gets a perfect and clear cut image on the retina, and without any effort, while the child with a troublesome hyperopia pursues its studies under the most annoying difficulties, frequently being accused of stupidity, and even chastised for what its teacher regards as indolence. The same rule in regard to debilitating diseases is also applicable to older persons. The accommodation power being thus reduced, no longer suffices to keep up a continuous adaptation, hence, the patient will date his eye trouble from such illness.

When any disease of the eye develops in consequence of an error of its refraction, it can not, as a rule, be permanently cured until all close work is given up, or the error corrected by suitable glasses. There are, however, exceptions to this rule.

I will now endeavor to outline the simplest method of determining hyperopia. It is not expected that the general practitioner is prepared with all the instruments and parapharnalia for such work. Indeed, they are not really necessary. With simply the test type of Snellen for distance, and Jerger for reading, and a comprehensive history of the case, you can, in nearly every instance, detect the error and approximate the degree.

Manifest hypermetropia. In this form, the degree is so high, with all the accommodation. The patient is unable to see clearly at any distance. The patient is asked to read the letters on the test type, which should be at least twenty feet distant, and in a good light. If, at that distance, he is unable to read the line marked 20, and can only read the line above, marked 30 or 40, he is either amblyopic, or has an error of refraction, and excluding the former, we are certain that he has a manifest hypermetropia of about 1, dioptric, or 40 inches, if he has read the line marked 30.

The first thought would be, the + 1 dioptric would be the glass for his correction, but such would rarely ever be the case, for only the manifest hypermetropia has been accounted for, and the amount of error hidden by the accommodation is still unknown. This latter can only be determined by the use of atropia. However, in most cases you would be justified in prescribing a + 2.30 D (26 inch), for reading. The question would most naturally here be, might this not be myopia? Yes; and a practical way to ascertain is to ask the patient to read Jerger number 1, at 4 or 6 inches; if he can not read it at that distance, or reads it imperfectly, it is not myopia. It is true, in high degrees of hypermetropia he might bring the letters quite close in order to enlarge retinal image, but the letters would be blurred, while in myopia they would be clear, black, and distinct.

In latent hypermetropia, that is, when the accommodation is able to overcome the error and vision $= \frac{20}{20}$ should you by previous symptoms suspect hypermetropia, the accommodation would have to be paralyzed with atropia. If, under the mydriatic v only $= \frac{20}{60}$, there would be a hypermetropia of about 3 D, (12 inches), and, in this case, instead of giving a still stronger glass, 1, to 1.50 dioptres would have to be deducted for the atropia.

DISCUSSION.

In regard to the remarks of Drs. Smith and Lott, I will say that I have devoted some time to this work of the refraction of school children, though, except for my own interest in gathering such data, it has come to nothing, for neither teachers nor parents have given it that importance which the subject demands.

